# An artificial transcription factor that activates potent interferon-γ expression in human Jurkat T Cells

**DOI:** 10.3389/fmmed.2024.1492370

**Published:** 2025-01-08

**Authors:** Ashley King, Davis Noblitt, Olivia Sherron, Clara Kjerfve, Lydia Pless, Nicholas L. Truex

**Affiliations:** ^1^ Department of Chemistry and Biochemistry, University of South Carolina, Columbia, SC, United States; ^2^ College of Engineering and Computing, University of South Carolina, Columbia, SC, United States

**Keywords:** transcription factor, interferon-gamma, enzyme-linked immunosorbent assay (ELISA), Jurkat, immunotherapy, protein design and engineering, next-generation sequencing, human leukocyte antigen

## Abstract

Interferon (IFN)-γ is a central regulator of cell-mediated immunity in human health and disease, but reduced expression of the target receptors impairs signaling activity and leads to immunotherapy resistance. Although intracellular expression of IFN-γ restores the signaling and downstream functions, we lack the tools to activate the *IFNG* gene instead of cell surface receptors. This paper introduces the design and characterization of an artificial transcription factor (ATF) protein that recognizes the *IFNG* gene with six zinc finger domains, which are dovetailed to a VP64 signaling domain that promotes gene transcription and translation. Biological studies with human Jurkat T cells reveal that the ATF amplifies *IFNG* gene transcription and translation, and also stimulates gene transcription for multiple class I and II HLA alleles and interferon-stimulated genes (ISGs). Biophysical characterization showed the recombinant ATF protein recognizes the human *IFNG* gene with nanomolar affinity (K_D_ = 5.27 ± 0.3 nM), adopts a protein secondary structure associated with the ββα-fold of zinc finger domains, and is resistant to thermal denaturation. These studies demonstrate that transcriptional targeting of cytokine genes, rather than surface receptors, activates cytokine expression and shows significant potential for directing immune function.

## Introduction

Cytokines and chemokines are important signaling proteins associated with immune cell development, activation, communication, differentiation, growth, and survival (Leonard and Lin, 2023). These proteins are typically classified as either pro- or anti-inflammatory molecules (Dinarello, 2000), but can also serve polyfunctional roles depending on the magnitude and timing of expression (Cui et al., 2024a). Tight regulation of cytokine genes is therefore vital to limit chronic inflammation or unwanted immune tolerance mechanisms, and to generate inflammation in response to infectious diseases and cancer (Leonard and Lin, 2023; Dinarello, 2000; Rauch et al., 2013).

Immunotherapies often regulate cytokine expression by targeting cell surface receptors with adjuvants and immune-checkpoint blockade inhibitors, but reduced expression of the target receptors impairs immune function. Moreover, achieving precise control of the magnitude and timing of cytokine expression remains an unmet challenge that limits therapeutic efficacy and safety (Cui et al., 2024a). Immunotherapies that seek to control cytokine genes, instead of targeting cell surface receptors, not only offer the promise of providing a powerful alternative means to control innate and adaptive immune pathways but also to improve cytokine expression across therapeutic, prophylactic, or tolerance-based immunotherapy platforms (Ghozikali et al., 2022; Grasso et al., 2020; Hosseinzadeh et al., 2024; Sriskandan et al., 1986).

Interferon (IFN)-γ is a key driver of innate and adaptive immune responses that is secreted by activated T lymphocytes, NK cells, B cells, dendritic cells (DCs), macrophages, and other immune cells. IFN-γ canonically exerts activity upon binding to receptors IFNGR1 and IFNGR2 to activate the Janus kinase (JAK)-signal transducer and activator of the transcription (STAT) pathway (Bach et al., 1997; Darnell et al., 1994; Tau and Rothman, 1999). Intracellular expression of IFN-γ has also been shown to induce an antiviral state in mammalian cells in a receptor-independent fashion (Sanceau et al., 1986). Additional functions of IFN-γ include the upregulation of major histocompatibility complex (MHC) class I and II molecules, which favors the priming of antigen-specific cytotoxic T lymphocytes (Castro et al., 2018; Schoenborn and Wilson, 2007), and also the activation and differentiation of cytotoxic T cells that promote cancer cell death (Zaidi and Merlino, 2011; Martinez-Lostao et al., 2015; Miller et al., 2009). These properties of IFN-γ are attractive for developing immunotherapies against cancer and infectious diseases, but systemic IFN-γ administration is ineffective due to rapid clearance and acute toxicity to healthy tissue (Rauch et al., 2013). Maintaining IFN-γ at localized concentrations through cell-to-cell communication dramatically improves therapeutic responses while limiting off-target toxicity (Gocher et al., 2022), and receptor-independent nucleus localization of IFN-γ is required to support intracellular signal transduction (Subramaniam et al., 2001; Bader and Weitzerbin, 1994; Subramaniam et al., 1999). To date, however, we have lacked intracellular tools to directly activate the *IFNG* gene instead of cell surface receptors.

**FIGURE 1:**
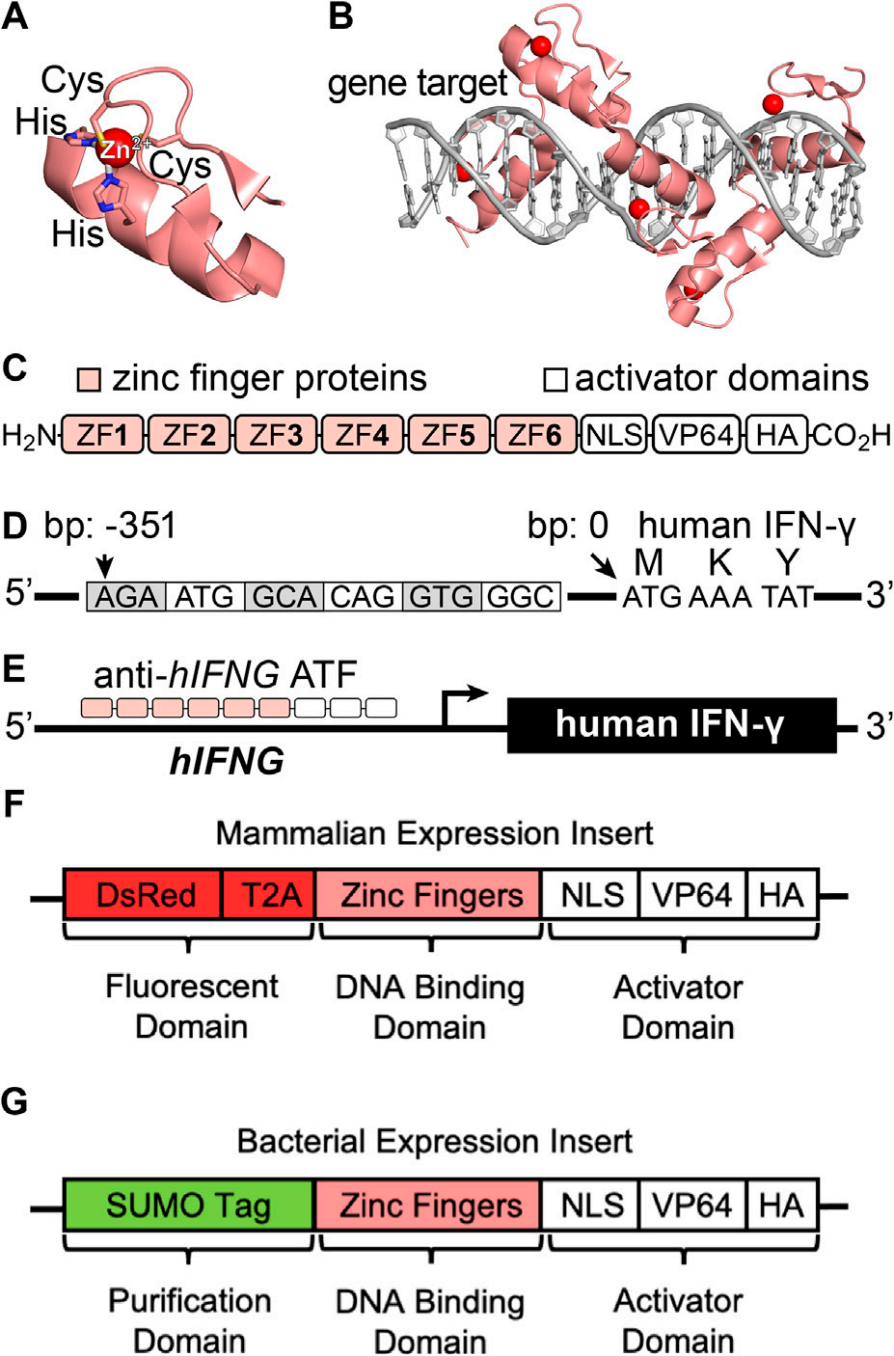
**(A, B)** Representative structures of a **(A)** zinc finger domain and **(B)** ATF recognition of the DNA gene target (PDB ID: 2I13). **(C)** Scheme of an artificial transcription factor (ATF) protein. **(D)** Illustration of the promotor region of the *hIFNG* gene (NCBI Gene ID: 3,458, GRCh38.p14, human chromosome 12), depicting the target base pairs (bp) of the IFN-γ promoter region relative to the start codon. **(E)** Illustration of ATF-mediated transcriptional activation and protein expression. **(F, G)** Gene-encoded ATF designs for **(F)** mammalian and **(G)** bacterial expression.

To improve the control of IFN-γ for research and therapeutic settings, we set out to develop a protein tool that activates intracellular expression at the transcriptional level. We developed an artificial transcription factor (ATF) that recognizes the human *IFNG* (*hIFNG*) gene and activates protein transcription and translation (Figure 1). The ATF design uses established zinc finger (ZF) proteins that provide programmable building blocks for gene recognition (Figures 1A, B) (Gaj et al., 2013; Negi et al., 2023). The ATF also contains a transcriptional activator domain that acts inside cells, rather than on cell surface receptors (Figures 1C–E). The ATF provides an attractive approach to activate the expression of IFN-γ and other immune signaling proteins within specific cells and tissues, particularly when paired with an appropriate intracellular delivery system.

## Materials and methods

### Construction of artificial transcription factor proteins

To identify *IFNG* gene regions for activating IFN-γ expression, human chromosome 12 was downloaded from the National Center for Biotechnology Information (NCBI, Gene ID: 3,458, annotation release: RS_2023_10). The targeted gene includes the 500 base pairs (bp) upstream of the first exon, which was imported into the ZFTools website for identifying predicted binding locations. The program was adjusted to identify 18 bp sequences that the ZF proteins could recognize, through dovetailing six individual ZF subunit proteins (Figures 1C,D). Four sets of ZF proteins were selected that bind throughout the IFN-γ promoter region (Supplementary Figure S1; Supplementary Tables S1–S3).

### Mammalian ATF expression plasmids

Genetic sequences for ATFs **1**–**4** were optimized for homo sapien codons and clonally inserted into a mammalian expression plasmid (pTwist CMV Puro, Twist Biosciences). A plasmid encoding only the DsRed fluorescent protein was also prepared as a transfection control. Another plasmid encoding a DsRed-Aart6 fusion protein was also prepared as an isotype control (Segal et al., 2006). The mammalian expression plasmids were transformed into *E. coli* (DH5-α, cat. no. C2987, New England Biosciences), expanded overnight in 1000-mL cultures at 37°C with Luria-Bertani (LB) broth and carbenicillin (100 μg/mL), and isolated by centrifugation. The cultures were subdivided for purification by the ZymoPURE II Maxiprep kit (Zymo Research, cat. no. D4203). Plasmids were eluted from the membrane with 2 × 200 µL (400 µL total) of ultrapure water (18 MΩ), which had been pre-filtered (0.4 µm) and warmed to 37°C. Isolated plasmids were further purified to remove endotoxin and stored at −20°C. DNA concentrations were determined based on the optical density at 260 nm using a SpectraDrop microplate (0.5 mm path length) and a UV-vis plate reader (SpectraMax iD3). The recombinant plasmids typically yielded ≥3 mg of rDNA per 1,000 mL of culture.

### Mammalian cell culture

Human Jurkat cells (ATCC, TIB-152) were cultured in Roswell Park Memorial Institute Medium (RPMI) containing GlutaMax supplemented with 10% v/v fetal bovine serum (FBS, Gibco) and 100 U/mL penicillin-streptomycin (Cytiva) at 37°C in 5% v/v CO_2_ (g).

### Plasmid transfection into Jurkat cells

ATF **1**–**4** plasmids were transfected into Jurkat T cells using a Bio-Rad Gene Pulser Xcell Electroporator. Before electroporation, the cells were grown to 80% confluency, collected by centrifugation (1,000 g × 5 min), manually counted on a hemacytometer, and resuspended to give a working stock solution of 5 × 10^6^ cells/mL in RPMI (10% FBS + Pen/Strep). To a 2 mm MicroPulser cuvette (Bio-Rad, cat. no. 1652086), 1 × 10^6^ Jurkat cells were added with 100 µg of plasmid in RPMI to reach a total volume of 200 µL. After gently mixing the suspension with a pipette, the transfection was performed using an exponential decay pulse; capacitance = 1,000 µF and voltage = 140 V. Four sets of electroporated cells (200 µL) were pooled into one well of a 6-well plate with complete RPMI media (final volume = 2 mL) and equilibrated for 1 h at 37°C and 5% CO_2_. The cells were further incubated in the presence of 10 µM ZnCl_2_, 1X phorbol 12-Myristate 13-Acetate (PMA)/ionomycin, and 3 μg/mL brefeldin A. At the indicated time points, the cells were processed and analyzed.

### Fluorescence microscopy

Transfection efficiency was determined using fluorescence microscopy based on the fraction of live cells expressing the DsRed (λ_Ex_ = 558 nm, λ_Em_ = 583 nm) protein. Cells were prepared for microscopy by transferring the cell suspensions into microcentrifuge tubes (1.7 mL), pelleting by centrifugation (1,000 × g, 5 min), and resuspending in PBS. Live-cell staining was achieved by incubating the cells with the Calcein AM viability dye (Invitrogen), pelleting by centrifugation, and resuspending in PBS. Microscopy images of Calcein AM (λ_EX_ = 494 nm, λ_EM_ = 517 nm) were obtained at ×5 and ×10 magnification and an exposure time of 300 msec. Images of DsRed-expressing cells were obtained at ×5 and ×10 magnification with an exposure time of 6.53 s. Transfection efficiencies were calculated using NIH ImageJ. Scale bars were calculated using the 1951 United States Air Force resolution test chart.

### ELISA

A sandwich ELISA protocol enabled the measurement of IFN-γ expression from Jurkat cells. High-binding 96-well plates (Corning, 9018) were treated with 100 µL of purified anti-human IFN-γ (0.5 mg/mL, BioLegend, 507502) and incubated at 4°C for 16 h. The wells were washed with PBS containing 0.05% Polysorbate-20 (3 × 100 µL) and blocked for 1 h with 200 µL of 1X the “Assay Diluent” buffer (BioLegend, 421203). Intracellular protein was isolated from the Jurkat cells after treatment with RIPA lysis buffer, quantified using a Bradford assay, diluted to 5 mg/mL concentration in the Assay Diluent buffer, and loaded in triplicate to the 96-well plate (100 µL each). Recombinant human IFN-γ (BioLegend, 570206) protein was also titrated into the 96-well plate to generate the standard curve, which ranged in concentration from 0 to 500 pg/mL. The plates were incubated for 2 h and washed with PBST (3 × 100 µL). Biotin anti-human IFN-γ (100 μL, 0.5 mg/mL, BioLegend, 502504) was added to the wells, incubated for 1 h, and washed with PBST (3 × 100 µL). HRP-Avidin (BioLegend, 405103) was added to the wells (100 µL), incubated for 30 min, and washed with PBST (3 × 10 µL). TMB substrate (BioLegend, 421501) was added to the wells (50 µL), incubated for 15 min, and quenched with 100 µL of stop solution (BioLegend, 423001). The absorbance of the wells was recorded at 450 nm, IFN-γ concentrations were calculated in Excel based on a standard curve with 0–500 pg/mL of IFN-γ protein. The experiment results were plotted and analyzed in GraphPad Prism. Statistical significance was evaluated using ANOVA with Holm–Sidak multiple comparisons test (*p < 0.05; **p < 0.005; ***p < 0.001 and ****p < 0.0001).

### RNA sequencing experiments

Cells obtained from the treatment conditions were processed using a RNeasy Minikit (Qiagen, 74106) and RNase-free DNase (Qiagen, 79254). The total RNA was collected in nuclease-free water and stored at −80°C until further analysis. The RNA was reverse-transcribed into double-stranded cDNA libraries by poly(A) selection using a NEBNext Ultra II RNA Library Prep Kit for Illumina (New England Biolabs, Ipswich, MA, United States), and sequenced using paired-end short-read sequencing (2 × 150 bp) on an Illumina platform (Azenta/Genewiz).

### RNA-seq data preprocessing

FASTQ files containing raw paired-end sequencing reads were processed using the CLC Genomics Workbench 24.0.2. Quality trimming steps included the removal of adapter sequences detected in reads. Reads with more than two ambiguous nucleotides were trimmed; sequences shorter than six nucleotides were discarded. Trimming also addressed low-quality sequences with a quality limit of 0.05. The resulting trimmed reads maintained an average length between 144.83 and 147.29 bases across samples. Approximately 13.9% of read pairs were trimmed due to adapter contamination. The final number of genes in the expression profile of all samples was 24,493.

The data analysis pipeline script was written in RStudio to analyze RNA-seq differential gene expression results; processing, statistical analysis, and visualization were conducted using specific R packages. The analysis required a raw gene expression counts matrix downloaded from the CLC Genomics Workbench and a metadata table containing sample-specific treatment information. Both files were uploaded in CSV format. A validation step ensured the metadata sample names matched the appropriate column names in the count matrix. The count matrix was reordered to align with the metadata as a reactive value for differential expression analysis. Raw fastq files of the data are available from the Gene Expression Omnibus (GEO) database under accession number GSE284420.

### Differential expression analysis

Differential expression analysis was performed using the DESeq2 package, which models count data using a negative binomial generalized linear model. The DESeq () function was used to estimate normalization factors, dispersion, and model parameters (Love et al., 2014). Log2 fold changes and associated statistics (standard errors, p-values, adjusted p-values) were calculated in redefined contrasts against the two controls, Aart6 and Shock. Wald tests were used for hypothesis testing, and adjusted p-values were calculated using the False Discovery Rate (FDR) method. Results for specific contrasts were extracted and exported as CSV files for further analysis and visualization. All scripts used to process and analyze the RNA-seq data may be found at https://github.com/TruexResearchLab/ATF_RNAsequencing2024.

### Data visualization

Principal Component Analysis (PCA) was used to explore variance structures in the gene expression data. Before PCA, genes with zero or constant variance were filtered, and the data was standardized. The analysis was performed using the prcomp () function, and the first three principal components were visualized in an interactive 3D scatterplot created with the plotly package. Volcano plots provided a graphical representation of the relationship between log2 fold changes and statistical significance, highlighting genes that surpassed defined thresholds (log2 fold change >1 or < −1, adjusted p-value <0.05), as indicated by vertical lines. Similarly, MA plots visualized the relationship between mean expression levels and log2 fold changes, with fold change thresholds (log2 fold change >1 or < −1, adjusted p-value <0.05) indicated by horizontal lines. Scatterplots were employed to compare test statistics between the experimental conditions, and genes meeting significance criteria (T-value >10 or < −10, on each axis) were highlighted and labeled. Heatmaps, generated using the pheatmap package, displayed log2-transformed expression levels of selected genes averaged across treatment groups, with row-wise scaling to emphasize relative expression changes. All visualizations were exported as PDFs. Boxplots of gene expression were generated using rlog-transformed data from DESeq2 results. Selected genes were used to subset the transformed expression matrix, and the data were combined with metadata for plotting. Boxplots were set by gene and included jittered sample points, with expression grouped by treatment.

### SDS-PAGE

Prior to electrophoresis, samples were mixed with loading dye, boiled (95°C, 5 min), and loaded into a polyacrylamide gradient (4%–16%) gel equilibrated with a Tris-Glycine SDS running buffer (Invitrogen). Gels were electrophoresed at 200 V for 40 min, visualized by Coomassie stain, and imaged using an Azure 600 Imaging System. Protein molecular weights were compared to a SeeBlue Plus2 (Invitrogen) molecular weight ladder.

### Mass spectrometry

Protein identity was confirmed by analytical LC-MS using an Agilent 1260 RP-HPLC coupled to an Agilent Q-TOF 6545XT. Proteins (100 ng) were injected from a 0.1 mg/mL sample of 50% CH_3_CN in H_2_O with 1% formic acid (FA). Samples were eluted with a gradient of 1%–61% CH_3_CN over 10 min on an Agilent Poroshell C18 column with a 0.5 mL/min flow rate. Spectra were recorded under positive ionization mode with an extended dynamic range (2 GHz) and standard mass range (m/z from 300 to 3,000).

### Bacterial ATF expression plasmids

The genetic sequence for SUMO-ATF **3r** was designed as a fusion protein (Supplementary Table S6) and codon-optimized for recombinant *E. coli* expression (Supplementary Table S7). The gene was clonally inserted into a pET-21 (+) bacterial expression plasmid (Twist Biosciences). The plasmid was transformed into *E. coli* (BL21, New England Biolabs, cat. no. C2527H), expanded into 1 L of LB with carbenicillin (100 μg/mL), and grown (37°C, 180 rpm) until an optical density (OD_600_) of 0.6–0.8 was reached. Protein expression was induced by the addition of IPTG (final conc. = 0.4 mM), and allowed to proceed overnight (30°C, 150 rpm). The cultures were isolated by centrifugation (20 min × 4,000 rpm) in 500-mL polypropylene centrifuge tubes (corning), transferred to a 50-mL conical tube, and stored at −80°C as frozen pellets.

The recombinant protein was purified after thawing and processing the frozen pellets. Pellets were resuspended in Tris-buffered saline (100 mM Tris, 500 mM NaCl, pH = 7.4) supplemented with an EDTA-free protease inhibitor and lysozyme (10 μg/mL). Cell suspensions were lysed by sonication and fractionated by centrifugation (12,100 rpm, 30 min). The supernatant was loaded onto a 5-mL Ni NTA (FF, Cytiva) column for affinity-based purification. Protein was eluted using a linear elution gradient with an imidazole buffer (10 mM Tris, 150 mM NaCl, and 500 mM imidazole, pH = 7.4). Samples at each stage of purification were collected for analysis by SDS-PAGE and LC-MS.

### Protein cleavage and purification

ATF **3r** was obtained after cleavage of the fusion protein. SUMO-ATF **3r** was transferred to tris-buffered saline (TBS) to ensure the removal of residual imidazole, treated with 1% Ulp-1 (w/w), incubated at 30°C, and monitored by LC-MS. ATF **3r** was purified on a Ni NTA column to remove the SUMO tag and Ulp-1. The identity of ATF **3r** was confirmed by SDS-PAGE and LC-MS.

### Preparative RP-HPLC

ATF **3r** protein was dissolved in a solution of 20% acetonitrile in water with 0.1% TFA, pushed through a syringe filter (Nylon, 0.22 µm), and purified by RP-HPLC using an Agilent 1,290 Infinity II Preparative Open-Bed Sampler/Collector. Protein was loaded onto an Agilent Prep-C18 column (21.2 × 50 mm, 5 µm particle size) at a flow rate of 15 mL/min, and eluted with a 10%–40% gradient (1% per minute) of solvent B in A (solvent A: water, 0.1% TFA; solvent B: acetonitrile, 0.1% TFA). Fractions of the eluted protein were collected and analyzed by LC-MS. Fractions containing the purified protein were pooled and lyophilized.

### Protein refolding

Lyophilized ATF **3r** protein was resuspended at 1 mg/mL in 10 mM HEPES buffer at pH 7.4. The solution was dialyzed with 10 mM HEPES to remove residual TFA. The cysteine residues were reduced by incubating with 6.67 mM TCEP (from a 20 mM stock solution) at 37°C for 1 h. The protein was subjected to refolding conditions by adding zinc acetate to a final concentration of 10 mM, followed by further incubation at 37°C for 1 h. The folded protein was obtained after dialysis with 10 mM HEPES to remove excess TCEP and zinc acetate.

### Circular dichroism

CD spectra of ATF **3r** were recorded in a 1-mm quartz cuvette on a J-1500 Jasco spectrometer at 185–260 nm (20 nm/min). Protein samples were prepared at 35 mM in 10 mM HEPES buffer at pH 7.4 and allowed to equilibrate to room temperature before analysis. Data were collected in triplicate and the signal was averaged to give the final CD spectrum.

### Differential scanning fluorimetry

Thermal stability of ATF **3r** was determined by heat denaturation experiments in the presence of a fluorophore. The protein was suspended at a concentration of 1 mg/mL in 10 mM HEPES (pH 7.4) and treated with an equal amount of SYPRO Orange (10X). Samples were evaluated on a Bio-Rad thermocycler by increasing the temperature with a linear ramp from 25°C to 90°C (rate = 0.5°C per min), collecting simultaneous fluorescence measurements of SYPRO Orange (λ_Ex_ = 491 nm, λ_Em_ = 586 nm). Data were background-subtracted in Excel and plotted in GraphPad Prism.

### Electrophoretic mobility shift assay

DNA recognition of ATF **3r** was evaluated upon mixing the protein with the target DNA sequence. Mixtures of protein and DNA were prepared at a fixed total volume (20 µL) and DNA concentration (20 pmol), but with increasing equivalents of the ATF **3r** protein (0–20).

The sample buffer comprised 2.5% glycerol, 5 mM MgCl_2_, 0.05% NP-40, 50 mM KCl, followed by the addition of nuclease-free water to achieve 20 µL. Samples were gently mixed, incubated at 0°C for 1 h, and treated with SDS-free loading dye. Samples were loaded into a 4%–8% native polyacrylamide (PAGE) gel (29:1) containing TBE buffer (0.045 M tris, 0.045 M borate, and 0.0 1M EDTA, pH 8.2–8.4), followed by separation at 100 V for 60 min at 5°C. The gels were visualized in a TBE buffer containing 1X SYBR Safe Stain and imaged using an Azure600 Imager. Band intensities were measured by NIH ImageJ, normalized in Excel, and plotted in GraphPad Prism.

## Results and discussion

### Design of artificial transcription factors

We designed the ATFs by obtaining the *hIFNG* region of human chromosome 12. The target region is 400 base pairs (bp) upstream of the IFN-γ start codon. Analysis of this region with the ZFTools program identified fusion ZF proteins based on previously reported binding affinities to DNA triplets (Supplementary Table S1). We selected sets of ZF proteins to incorporate into ATFs, called ATF **1**–**4** (Supplementary Tables S2, S3). The ATFs contain six-linked ZF proteins that recognize 18-bp regions throughout the *hIFNG* gene (Beerli et al., 2000; Liu et al., 1997).

ATFs **1–4** comprise three protein domains to evaluate transcription factor activity (Heiderscheit et al., 2018), each with a distinct function: fluorescence, gene targeting, and transcriptional activation (Figure 1F; Supplementary Tables S4, S5). The fluorescent domain encodes Discosoma red (DsRed) for monitoring intracellular localization by fluorescence microscopy (Gross et al., 2000; Beier and Gross, 2006; Piatkevich and Verkhusha, 2010). The gene targeting domain comprises the zinc finger proteins that recognize the *hIFNG* gene.

The ATFs encode a transcriptional activation domain with three signals: a nuclear localization signal (NLS), four tandem copies of virus protein 16 (VP64), and a hemagglutinin A (HA) tag. NLS directs the ATF to the nucleus to access the intracellular genes (Lange et al., 2007). VP64 is a coactivator that facilitates transcription and translation (Maeder et al., 2013). HA is included for protein tagging (Margine et al., 2013). Altogether, the sequences for ATFs **1**–**4** were codon optimized for expression in human cells (Supplementary Table S4), and incorporated into a mammalian expression vector (pTwist) with a CMV promoter. For biophysical studies, we also developed a sequence for recombinant expression in bacterial cells (Figure 1G).

### Evaluating ATF activity in human Jurkat T Cells

**FIGURE 2:**
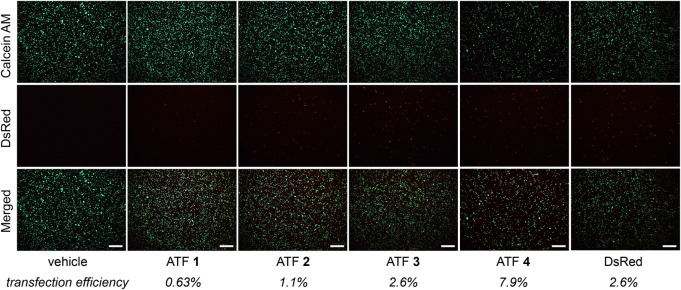
Fluorescence microscopy panel after ATF **1**–**4** transfections with Jurkat cells. Left-hand text indicates images for viability (Calcein AM) and transfection efficiency (DsRed). The merged images represent an overlay of Calcein AM and DsRed images. Average transfection efficiencies are reported below each treatment (n = 3); scale bars = 250 µm.

The biological activity of the mammalian ATFs was evaluated in human Jurkat T cells. Although plasmid delivery into T cells is notoriously challenging (Chicaybam et al., 2013), successful transfection was achieved using electroporation. Figure 2 shows fluorescent images of the Jurkat cells, which indicate the transfection efficiency (DsRed) and cell viability (Calcein AM). Transfection efficiency was determined by comparing the red fluorescent cells with the total viable cells (Schneider et al., 2012). The transfection was successful for all plasmids but varied slightly for each ATF. Upon confirming that the cells produce the DsRed protein, we proceeded to evaluate each ATF and the effects on IFN-γ expression.

### ATF-mediated IFN-γ production

Human Jurkat T cells produce minimal quantities of IFN-γ under steady-state conditions (Hu et al., 2002). Although human patients typically exhibit IFN-γ concentrations of 1 pg/mL (Kurtovic and Halilovic, 2018), treatment with PMA and ionomycin typically provides potent T cell activation by upregulating protein kinase C and facilitating the transfer of Ca^2+^ ions, respectively (Ai et al., 2013).

**FIGURE 3:**
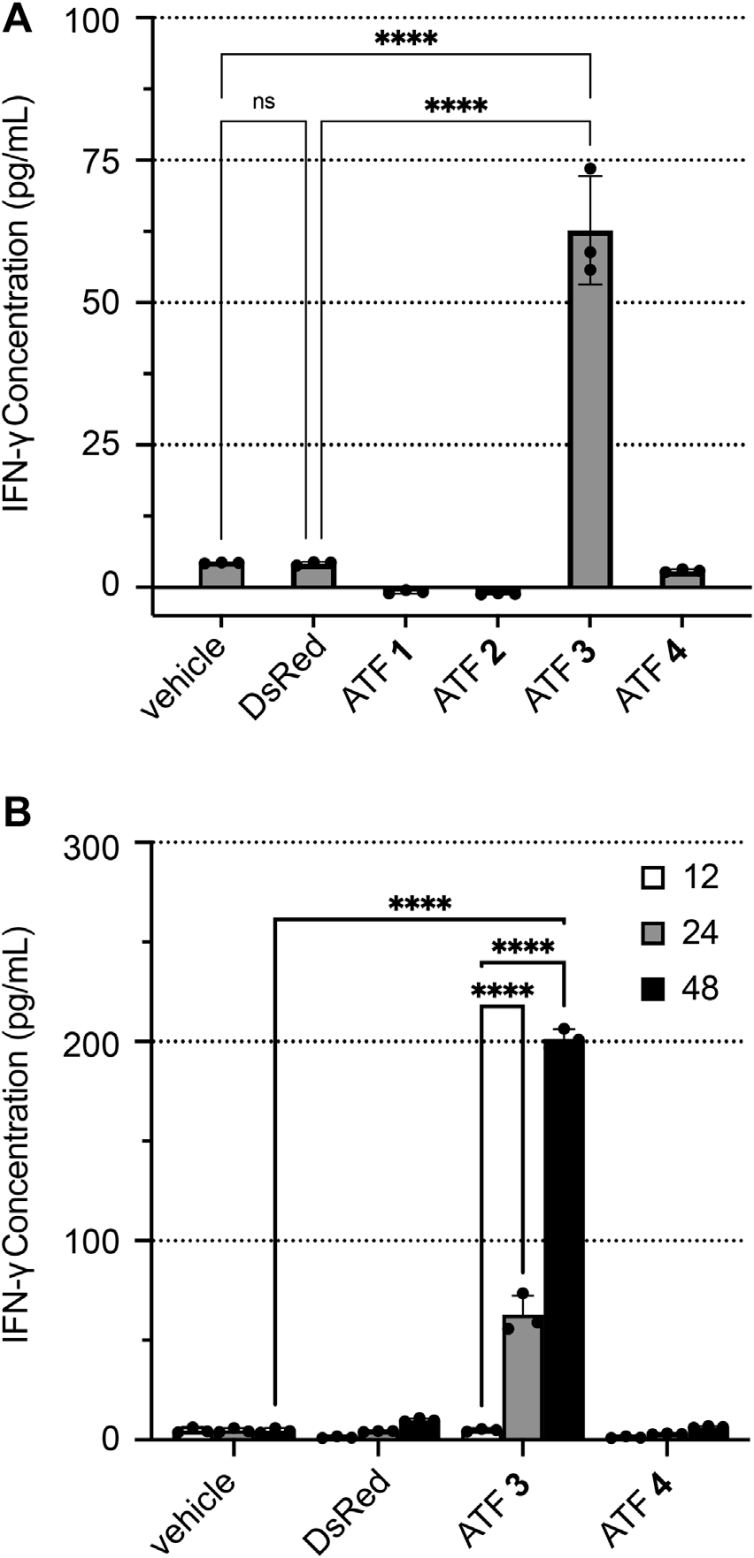
ATF activation of IFN-γ expression in human Jurkat T cells. IFN-γ concentrations were determined by ELISA after transfecting Jurkat cells with buffer (vehicle), plasmid-encoded DsRed, or plasmid-encoded ATFs **1**–**4**, followed by incubation with PMA/ionomycin, 3 μg/mL of brefeldin A (BFA), and 10 µM ZnCl_2_. Plots show IFN-γ concentrations after **(A)** 24 h and **(B)** time-course monitoring at 12, 24, and 48 h. Data represent the mean value of three replicate wells ±standard deviation (SD). Statistical significance was evaluated by ANOVA analysis with a Holm–Sidak test to compare each ATF treatment versus the ‘vehicle’ treatment (*p < 0.05; **p < 0.005; ***p < 0.001; and ****p < 0.0001).

The activity of each ATF was established in human Jurkat T cells through the measurement of IFN-γ expression using enzyme-linked immunosorbent assays (ELISAs). ATF **1**–**4** constructs were evaluated for their ability to mediate IFN-γ expression compared to the vehicle (buffer only) and DsRed plasmid (Figure 3A). The treatment with ATFs **1**, **2,** and **4** showed low levels of IFN-γ, indicating that these ATF constructs do not activate IFN-γ transcription and translation. Intriguingly, ATFs **1** and **2** appear to decrease IFN-γ expression, which may reflect the inhibition of the *hIFNG* gene. Treatment with ATF **3** increased IFN-γ levels to 62.7 pg/mL after a 24 h incubation, which is about a 14-fold increase compared to baseline levels. Increased IFN-γ expression by ATF **3** is also sustained over time (up to 48 h), which indicates that transcriptional activation is not an artifact from the transfection conditions (Figure 3B).

Two negative controls were tested to establish baseline IFN-γ production levels: (1) electroporated cells in the absence of plasmid, which is termed “vehicle”; (2) an empty plasmid that encodes DsRed without an ATF. Treatment of Jurkat cells with the “vehicle” and DsRed generated only 4.2–4.3 pg/mL of IFN-γ, indicating that neither condition significantly increases IFN-γ expression.

Additional testing of ATF activity further determined the baseline levels of IFN-γ production in Jurkat cells (Supplementary Figure S2). Incubating Jurkat cells with PMA/ionomycin showed no increase in IFN-γ production, indicating that PMA/ionomycin treatment alone does not increase IFN-γ expression. Brefeldin A (BFA) inhibits vesicle formation and halts protein export, allowing for accurate measurement of IFN-γ from the cell lysate (Chardin et al., 1999). Incubating the cells with BFA alone also showed no increase in IFN-γ production. The folding of ZF proteins in the ββα-fold conformation is stabilized by zinc ions. To assess the role of zinc ions for ATF activity (Supplementary Figure S3), ATF-transfected Jurkat cells were incubated with ZnCl_2_ (10 µM) for 24 h. These groups showed marginally higher IFN-γ expression than cells without ZnCl_2_, demonstrating that zinc ions play a role in supporting ATF activity.

### Activation of interferon-stimulated genes

**FIGURE 4:**
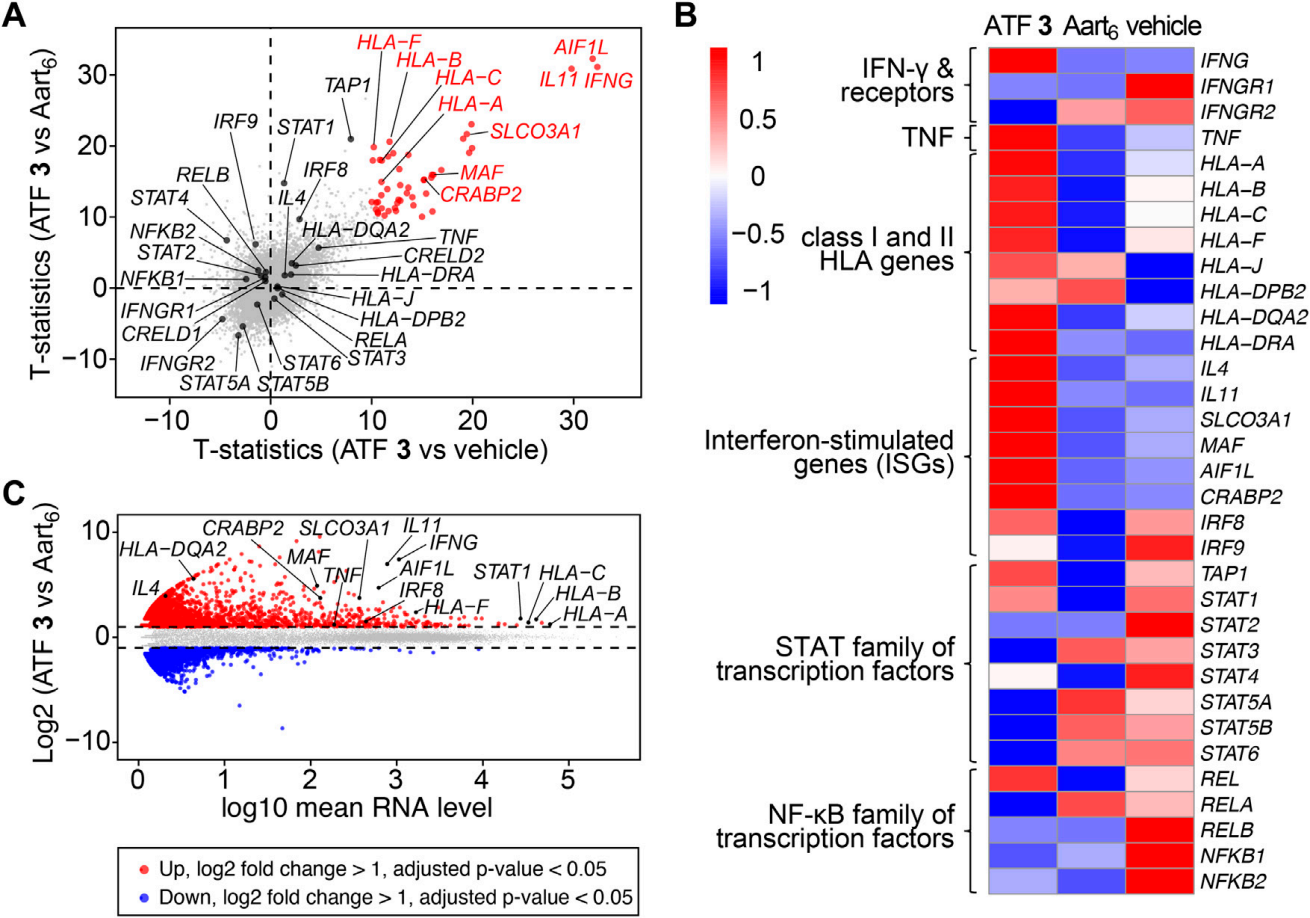
Artificial transcription factors activate *IFNG* and interferon-stimulated genes. Jurkat cells were transfected with ATF **3**, Aart_6_, or vehicle, and incubated for 48 h with PMA/ionomycin, 3 μg/mL of brefeldin A (BFA), and 10 µM ZnCl_2_. Total RNA was extracted and sequenced. Data represent the mean value of three replicate samples. Labels indicate *IFNG* and upregulated genes associated with human leukocyte antigens (HLAs) and interferon-stimulated genes (ISGs). **(A)** Scatterplot of t-statistics comparing the differences in gene transcription levels between ATF **3** versus (vs.) vehicle (*X*-axis) and ATF **3** vs. Aart_6_ (*Y*-axis). **(B)** Heatmap displaying the log-transformed values from the comparison across three samples, highlighting differentially expressed genes. **(C)** MA plot showing differentially expressed genes (DEGs) from ATF **3** compared to Aart_6_. Upregulated genes with adjusted p-value <0.05 and log2 fold change >1 are shown in red, and downregulated genes with p-value <0.05 and log2 fold change <1 are shown in blue.

The biological response from ATF **3** was assessed based on the transcriptomic profile of ATF-treated Jurkat cells (Cui et al., 2024b; Der et al., 1998). Due to the significant expression of IFN-γ observed for ATF **3**, we anticipated that this construct would also induce upregulation of the *IFNG* gene and associated interferon-stimulated genes (ISGs). We determine this activity by evaluating RNA-sequencing (RNA-seq) results from Jurkat cells transfected ATF **3**, and comparing these results to cells transfected with buffer ‘vehicle’ and a homologous ATF called Aart_6_. Differential gene expression (DGE) analysis of the total RNA showed upregulated expression of the target *IFNG* gene in cells treated with ATF **3**, indicating a successful activation of the target gene (Figure 4).

Signal transduction from IFN-γ stimulates the expression of class I and II human leukocyte antigens (HLAs), (van den Elsen et al., 1998), and also the STAT and IFN regulatory factor (IRF) families of transcription factors (Der et al., 1998). In Jurkat cells treated with ATF **3**, the transcriptomic profile shows upregulated expression of *STAT1* and *HLA* genes, including *HLA-A*, *HLA-B*, *HLA-C*, *HLA-DRA*, and *HLA-F*. The transcriptomic profile also shows upregulated genes for inflammatory proteins, including *IL11* (IL-11) and *AIF1L* (allograft inflammatory factor 1), and for NF-κB proteins, including *SLCO3A1* (solute carrier organic anion transporter family member 3A1), *MAF* (musculoaponeurotic fibrosarcoma), and *CRABP2* (cellular retinoic acid-binding protein 2). The upregulation of these genes supports the observation that ATF **3** mediates IFN-γ expression and, in turn, promotes signal transduction for inflammatory responses.

### Preparation of a recombinant artificial transcription factor

We isolated a recombinant form of ATF **3**, called ATF **3r**, to enable biophysical characterization of the protein. ATF **3r** was expressed as a fusion protein termed SUMO-ATF **3r**, purified by Ni NTA affinity chromatography, and treated with the Ulp1 cysteine protease. ATF **3r** was obtained after RP-HPLC purification and lyophilization, which gave the protein in pure form as a dry powder (trifluoroacetic acid salt). The identity of the protein was confirmed by SDS-PAGE and LC-MS (Supplementary Figures S4–S7), and further characterized to evaluate DNA recognition, protein folding, and thermal stability.

### Assessing ATF recognition of the target *IFNG* gene

**FIGURE 5:**
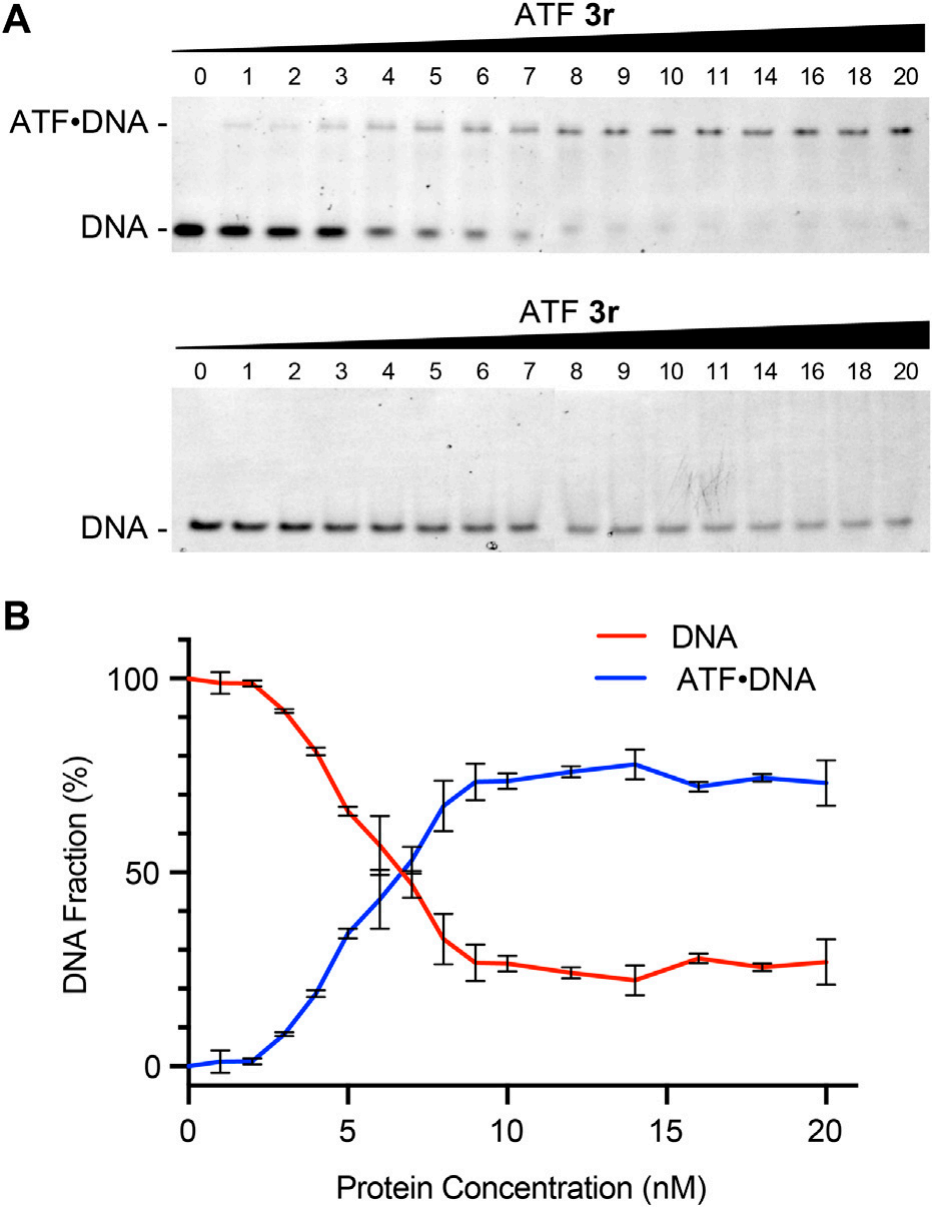
ATF protein recognition of the *hIFNG* gene. **(A)** Native PAGE analysis of mixtures containing ATF **3r** and synthetic double-stranded DNA (dsDNA) encoding *IFNG* (top) or an irrelevant sequence (bottom). **(B)** Plot showing the percentages of protein concentration (nM) versus free DNA or duplexed ATF**-**DNA. Data from ATF **3r** forming duplexed ATF-DNA was fitted to a 1:1 binding model, which showed an apparent K_D_ of 5.2 ± 0.3 nM. K_D_ was determined by a nonlinear fit of specific binding with Hill slope.

ATF recognition of DNA strands is mediated by the ZF proteins. To evaluate the gene recognition properties of ATF **3r**, we used electrophoretic mobility shift assays (EMSAs) to determine the binding selectivity, affinity, and stoichiometry (Figure 5). Prior binding studies of other zinc finger proteins containing six subunits have shown K_D_ values that range from 2 to 15 nM (Dreier et al., 2001). ATF **3r** contains six subunits but also an activation domain. Nonetheless, the EMSA studies show that ATF **3r** maintains effective and selective binding to the target region.

We evaluated the DNA-binding properties with synthetic double-stranded (dsDNA) fragments encoding the target *IFNG* sequence and an irrelevant sequence as a negative control. ATF **3r** protein was incubated with each DNA fragment, followed by separation with native PAGE and visualization with a SYBR Safe DNA stain (Figure 5A). Mixtures of ATF **3r** with the target DNA showed a second row of bands appear at higher protein concentrations, corresponding to a 1:1 ATF:DNA complex. Furthermore, the band intensities from the free DNA decrease at higher protein concentrations. Integration of the DNA band intensities shows that 50% is bound to protein at a 6:1 ratio of ATF **3r** to DNA (Figure 5B). The fraction bound was further plotted to approximate a K_D_. Fitting the data to a 1:1 binding model showed an apparent K_D_ of 5.2 ± 0.3 nM. This K_D_ is comparable with previously reported values of zinc finger proteins and establishes that ATF **3r** binds within the known range of six-linked zinc finger proteins. To further demonstrate the selectivity of ATF **3r**, the protein was mixed with an irrelevant DNA strand and evaluated (Figure 5A). EMSA showed a single set of horizontal bands that correspond only with free DNA, indicating that ATF **3r** does not recognize the irrelevant DNA sequence.

### Assessing ATF folding and thermal stability

**FIGURE 6:**
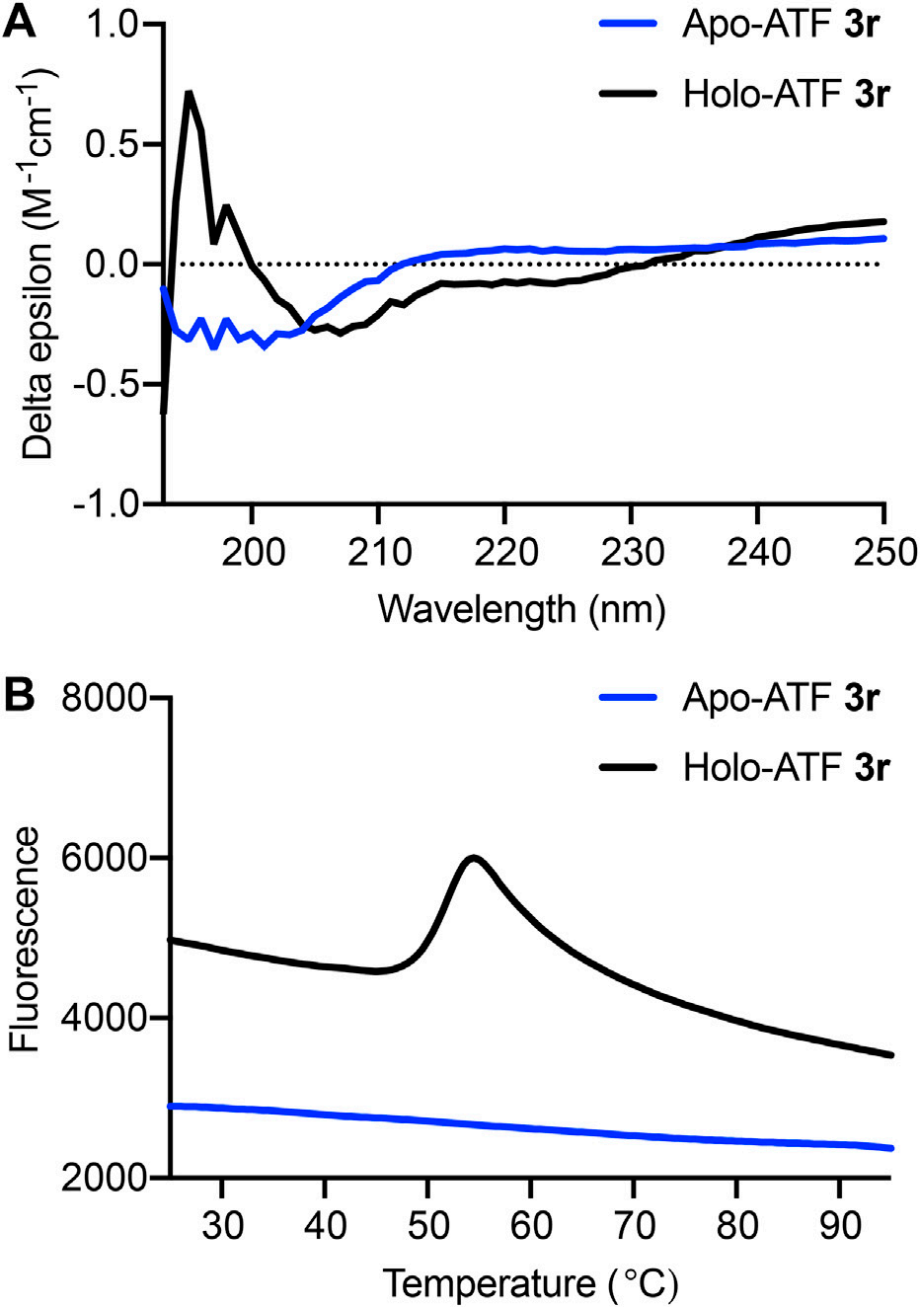
**(A)** Circular dichroism (CD) spectra from 190 to 250 nm show apo-ATF **3r** (blue) and holo-ATF **3r** (black). **(B)** Thermal stability of ATF **3r**. Differential scanning fluorimetry (DSF) of apo- and holo-ATF **3r**. First derivative of relative fluorescence, indicating the protein melting point (T_m_ = 52°C).

Circular dichroism (CD) and differential scanning fluorimetry (DSF) experiments were performed to evaluate ZF folding and resistance to thermal denaturation (Hajdu et al., 2023). These experiments established that ATF **3r** adopts a folded structure that is stable under biological temperatures (Figure 6). The zinc finger sequences within each ATF belong to the Cys_2_His_2_ family, which is characterized by adopting a ββα fold that is stabilized by a zinc ion (Razin et al., 2012). Performing CD analysis on the apo- and holoprotein provides valuable information on protein folding. CD analysis shows significant differences between ATF **3r** folding in the apo- and holo-forms (Figure 6A). Apo-ATF **3r** represents an unfolded protein with a negative peak centered around 200 nm, corresponding to random coils. Holo-ATF **3r** depicts a folded protein that has ββα characteristics. Folding of holo-ATF **3r** is demonstrated by the negative peaks at 205 and 220 nm, along with a positive peak at 200 nm. The protein characteristics depicted in the CD data are comparable to previous studies on similar zinc finger proteins (Hajdu et al., 2023).

Thermal stability is an important feature when considering therapeutic applications under biological conditions (Boivin et al., 2013; Pantoliano et al., 2001). DSF quantifies thermal stability by providing a melting temperature (T_m_) that can be compared to other proteins. Beyond determining the thermal stability of ATF **3r**, we also show that zinc ions are necessary for ATF protein folding. The T_m_ was determined by obtaining the first derivative of the DSF spectra and identifying the point of inflection (Figure 6B). The DSF analysis shows that the holo-ATF **3r** exhibits a T_m_ of 52°C, but apo-ATF **3r** does not adopt a folded structure.

## Conclusion

This study introduces a new transcription factor protein that enables transcriptional control of cytokine expression, rather than targeting cell surface receptors. This was accomplished through the design and characterization of an ATF protein that regulates the transcription and translation of endogenous IFN-γ. We report that ATF **3** successfully increases IFN-γ expression by about 14-fold and also induces transcriptional activation of *IFNG*, class I and II *HLA* alleles, and numerous inflammasome genes. Biophysical studies of the recombinant ATF **3r** protein establish zinc-dependent folding, thermal stability, and DNA recognition activity. ATF **3** and **3r** show significant potential as genetic or biomolecule approaches for modulating IFN-γ production. We envision that ATF activation will provide superior control of IFN-γ activity against cancer cell lines, particularly in the context of co-administering IFN-γ with a chemotherapy agent that have previously been shown to increase patient survival (Windbichler et al., 2000). IFN-γ expression is also known to directly correlate with cancer patient treatment outcomes and to exert potent antiviral properties.

Moreover, our studies show promise for using ATFs to improve immunotherapies through modulating other classes of immune signaling proteins. For targeting immune genes, ATFs can be easily reprogrammed to target additional chemokines, cytokines, and interleukins. The gene-encodable feature of ATFs is also readily translatable for integration and delivery with established immunotherapy and other therapeutic platforms, including RNA and DNA vaccines, adoptive cell-transfer therapies, and recombinant fusion proteins.

The research described in this paper represents a larger body of ongoing research in our laboratory. We envision that ATF-based immunotherapies readily enable control of pro- or anti-inflammatory signals against viruses, bacteria, and cancer, or even induce tolerance for treating autoimmune and transplant diseases. We further envision that ATFs can enable the reprogramming of tumor microenvironments to activate the expression of tumor antigens present at low levels or low mutational burdens. Libraries of ATFs could even facilitate the amplification of antigenic peptides to facilitate antigen presentation and recognition, including for latent oncovirus infections and also for tumor antigens and neoantigens associated with melanoma, glioblastoma, colon, triple-negative breast cancer, and other resistant cancers. Rapid automation of ATF development could even enable the activation of patient-specific tumor neoantigens to improve treatment outcomes for personalized cancer and other immunotherapies.

## Data Availability

The transcriptomic data generated in this study are publicly available. Raw fastq and processed data files are available from the Gene Expression Omnibus (GEO) database under accession number GSE284420. Code availability: All scripts used to process and analyze the transcriptomic data may be found at https://github.com/TruexResearchLab/ATF_RNAsequencing2024.
