# Supplementary material for: An artificial transcription factor that activates potent interferon-γ expression in human Jurkat T Cells
Source: Front Mol Med. 2025 Jan 8;4:1492370. doi: 10.3389/fmmed.2024.1492370 (PMC11751033; doi:10.3389/fmmed.2024.1492370)
Supplement: Supplementary file 1 [file Supplementaryfile1.pdf]

Supplementary information for

An Artificial Transcription Factor that Activates Potent Interferon-γ Expression in Human Jurkat T Cells

Ashley King[1], Davis Noblitt,[1] Olivia Sherron[1,2], Clara Kjerfve,[1] Lydia Pless[1], and Nicholas L. Truex[1]*

[1] Department of Chemistry and Biochemistry, University of South Carolina, 631 Sumter Street, Columbia, South Carolina, 29208, USA.

[2] College of Engineering and Computing, University of South Carolina, 301 Main Street, Columbia, South Carolina, 29208, USA.

* To whom correspondence should be addressed: ntruex@sc.edu

I. Supplementary Figures

Supplementary Figures 1. DNA promoter region of the *hIFNG* gene	3
Supplementary Figures 2. IFN-γ ELISA after PMA/ionomycin treatment of Jurkat cells	4
Supplementary Figures 3. IFN-γ ELISA after ATF transfection with zinc chloride	5
Supplementary Figures 4. Protein purification of SUMO-ATF **3r**	6
Supplementary Figures 5. Proteolytic Ulp-1 cleavage of SUMO-ATF **3r**	7
Supplementary Figures 6. Purification of cleaved ATF **3r**	7
Supplementary Figures 7. LC-MS (ESI) analysis of ATF **3r**	8

II. Supplementary Tables

Supplementary Table 1. Target nucleotide sequences in the promoter of *IFNG* gene	9
Supplementary Table 2. Variable amino acids for gene recognition by ZF subunits	9
Supplementary Table 3. Protein scaffold for dovetailing six zinc finger subunits	9
Supplementary Table 4. Amino Acid sequences for mammalian expression of ATF **1**–**4**	10
Supplementary Table 5. Amino Acid sequences for the subcomponents of ATF **1**–**4**	11
Supplementary Table 6. Amino Acid sequence for SUMO-ATF **3r**	12
Supplementary Table 7. Nucleotide sequence for SUMO-ATF **3r**	12

I. SUPPORTING FIGURES

-426
GCGAAGTGGGGAGGTACAAAAAAATTTCCAGTCCTTGAATGGTGTGAAGTAAAAGT
ATF **4**

-370
GCCTTCAAAGAATCCCACCAGAATGGCACAGGTGGGCATAATGGGTCTGTCTCATCG
ATF **3**

-313
TCAAAGGACCCAAGGAGTCTAAAGGAAACTCTAACTACAACACCCAAATGCCACAA
ATF **2**

-257
AACCTTAGTTATTAATACAAACTATCATCCCTGCCTATCTGTCACCATCTCATCTTAA

-199
AAAACTTGTGAAAATACGTAATCCTCAGGAGACTTCAATTAGGTATAAATACCAGC
ATF **1**

-143
AGCCAGAGGAGGTGCAGCACATTGTTCTGATCATCTGAAGATCAGCTATTAGAAGA

-87
GAAAGATCAGTTAAGTCCTTTGGACCTGATCAGCTTGATACAAGAACTACTGATTTC

-30 0 +15
AACTTCTTTGGCTTAATTCTCTCGGAAACG **ATG AAA TAT ACA AGT**
M K Y T S

Supplementary Figure 1. DNA promoter region of the *hIFNG* gene. Gene sequence shows the IFN-γ promoter region from -426 to +15 base pairs, which was obtained from the National Center for Biotechnology Information (NCBI) from gene ID: 3458, assembly: GRCh38.p14, annotation release: RS_2023_10. Blue text indicates the *hIFNG* start codon and subsequent four codons, and also indicates the five N-terminal amino acids of IFN-γ. Red text indicates the 18 base pair target sequences of ATFs **1–4**.

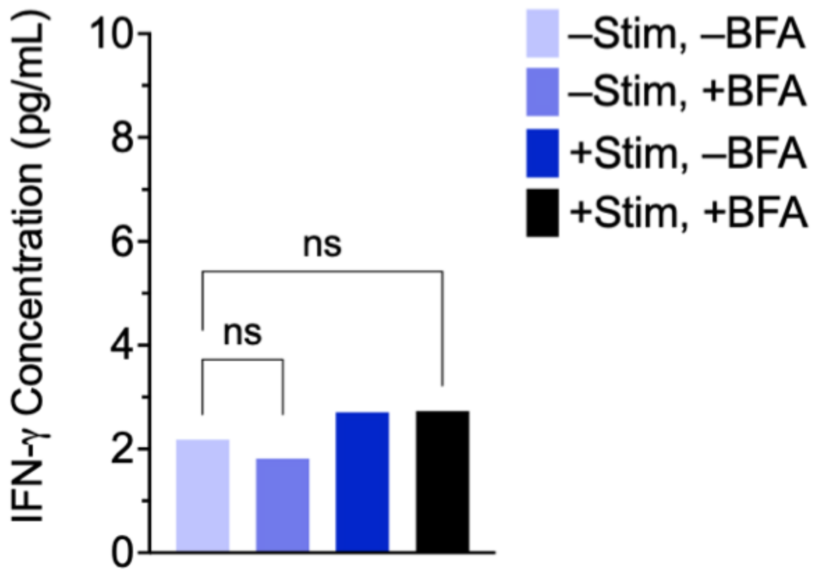

Supplementary Figure 2. Quantification of PMA/ionomycin (stim) induced activation of IFN-γ by enzyme-linked immunosorbent assay (ELISA), with (+) and without (–) brefeldin A (BFA) treatment. A sandwich ELISA was utilized to quantify IFN-γ from cell lysate. Protein content was quantified by Bradford protein assay; 5 mg/mL of total protein was loaded in each well. IFN-γ concentration was calculated based on a standard curve with recombinant protein. Statistical significance was evaluated by an ordinary one-way ANOVA analysis with Tukey's multiple comparisons with a single pooled variance to compare each treatment to the untreated (– Stim, – BFA).

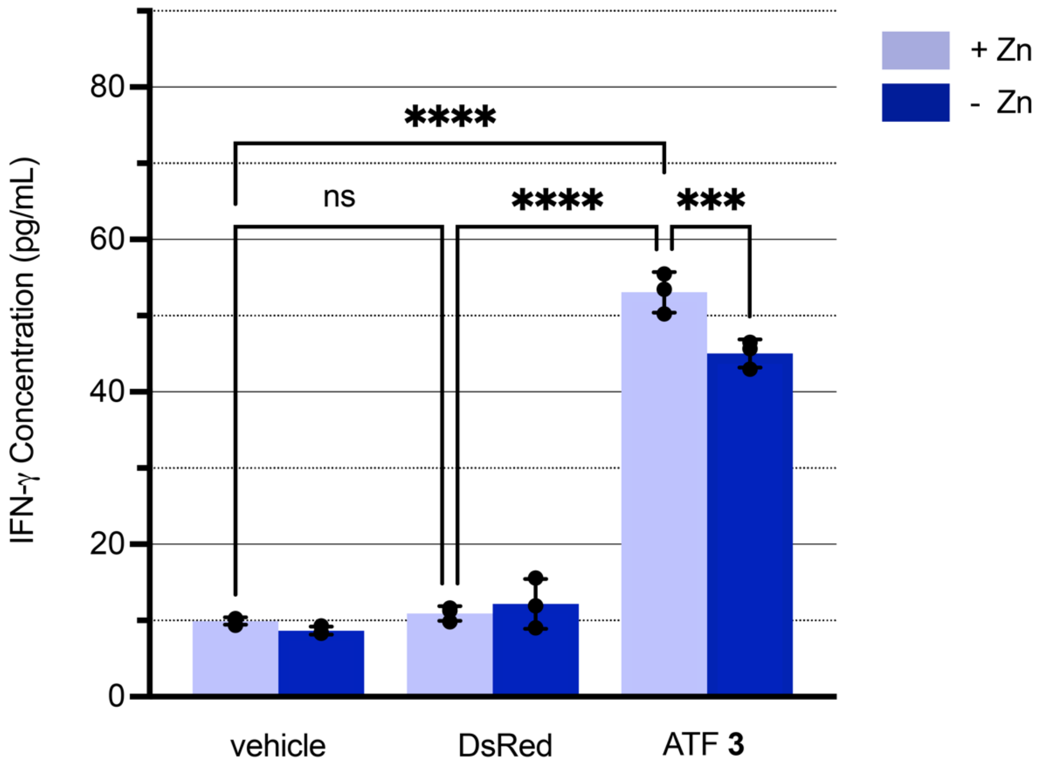

Supplementary Figure 3. ELISA measuring IFN-γ from immortalized human Jurkat T cells in the presence of zinc ions (Zn). Cells were electroporated using the indicated ATF plasmid, with (+) or without (–) 10 µM $ZnCl_2$. Cells were treated with lysis buffer at 24 h. Protein content was quantified by Bradford protein assay; 5 mg/mL of total protein was loaded in each well. IFN-γ concentration was calculated based on a standard curve recombinant protein. Data represent the mean value of three replicate wells ± standard deviation (SD). Statistical significance was evaluated by an ordinary one-way ANOVA analysis with Tukey's multiple comparisons with a single pooled variance to compare ATF treatment versus the 'vehicle' treatment without plasmid (*p< 0.05; **p< 0.005; ***p< 0.001 and ****p< 0.0001).

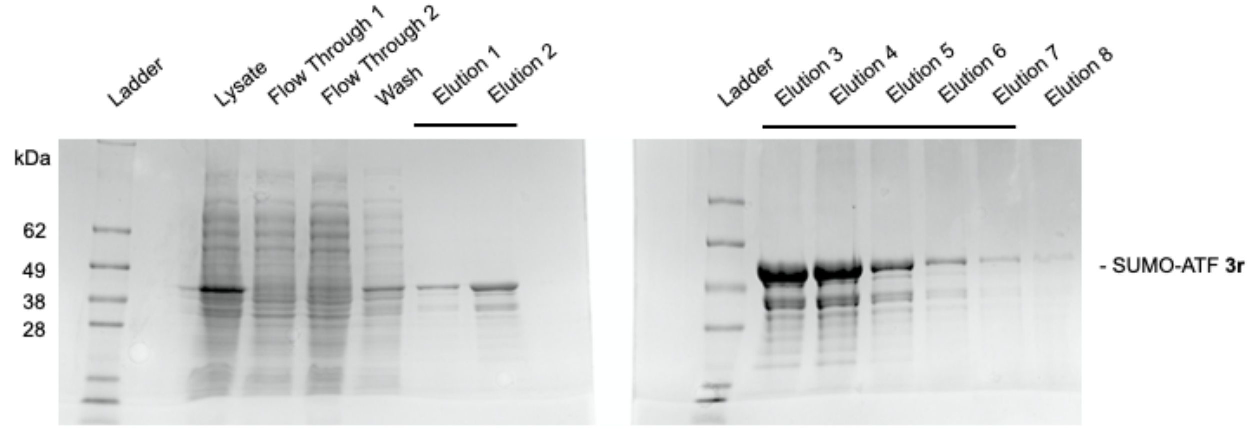

Supplementary Figure 4. Purification of SUMO-ATF **3r** from bacterial expression (*E. coli*). Coomassie-visualized SDS-PAGE of fractions from bacterial cell lysis, followed by Ni NTA purification. The indicated fractions were collected and pooled.

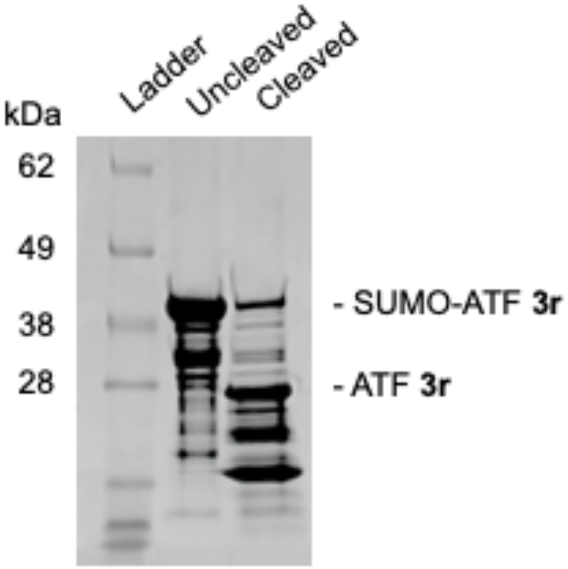

Supplementary Figure 5. Cleavage of SUMO-ATF **3**r by ulp-1. SUMO-ATF **3r** was treated with ulp-1 at a 100:1 ratio, incubated at 30 ºC for 1 h, and monitored by Coomassie-visualized SDS-PAGE.

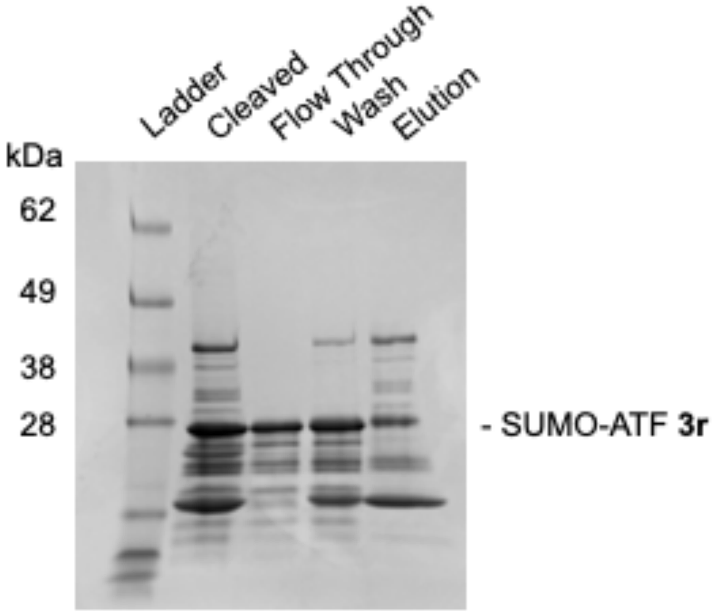

Supplementary Figure 6. Purification of ATF **3r**. Coomassie-visualized SDS-PAGE of fractions collected from Ni NTA purification after SUMO cleavage of ATF **3r**.

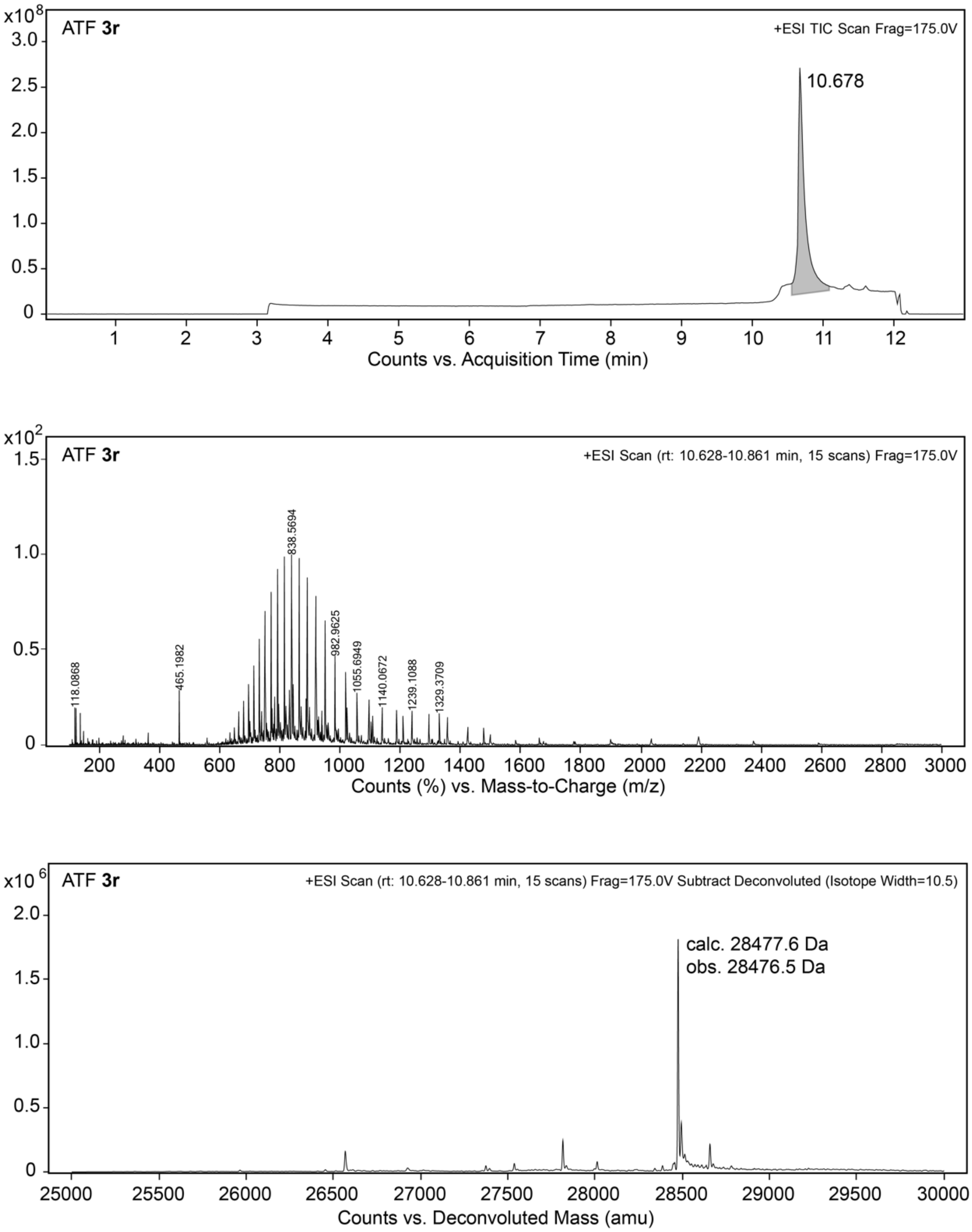

Supplementary Figure 7. LC-MS characterization of ATF **3r.** The protein was eluted over 10 min from an Agilent Poroshell C18 column, with a 0.5 mL/min flow rate and a gradient of 1–61% CH_3CN containing 0.1% formic acid. Electrospray ionization (ESI) mass spectrometry was used to detect the protein ions (m/z) and determine the deconvoluted mass (amu).

II. SUPPLEMENTARY TABLES

Supplementary Table 1. Target nucleotide sequences in the promoter region of the *IFNG* gene.

	ATF **1**	ATF **2**	ATF **3**	ATF **4**
gene target	CTTGTGAAAATACGTAAT	GAAACTCTAACTACAACA	AGAATGGCACAGGTGGGC	ATGGTGTGAAGTAAAAGT

Supplementary Table 2. Variable amino acids for gene recognition by ZF subunits.

	ATF **1**	ATF **2**	ATF **3**	ATF **4**
ZF 1	TTGNLTV	SPADLTR	DPGHLVR	HRTTLTN
ZF 2	SRRTCRA	SPADLTR	RSDELVR	QRANLRA
ZF 3	QKSSLIA	THLDLIR	RADNLTE	HRTTLTN
ZF 4	QRANLRA	QNSTLTE	QSGDLRR	QAGHLAS
ZF 5	RSDELVR	THLDLIR	RRDELNV	RSDELVR
ZF 6	TTGALTE	QSSNLVR	QLAHLRA	RRDELNV

Supplementary Table 3. ATF protein scaffold showing the amino acid sequences for six zinc finger (ZF) subunits with variable regions for targeted gene recognition.

Component	Amino Acid Sequence
ZF	LEPGEKPYKCPECGKSFSXXXXXXXHQRTHTGEKPYKCPECGKSFSXXXXXXXHQRTHTGEKPYKCPECGKSFSXXXXXXXHQRTHTGEKPYKCPECGKSFSSXXXXXXHQRTHTGEKPYKCPECGKSFSXXXXXXXHQRTHTGEKPYKCPECGKSFSXXXXXXXHQRTHTGKKTS
[1] Underlined amino acids, shown as 'XXXXXX', indicate the variable regions for targeted gene recognition.	

Supplementary Table 4. Amino acid sequences for mammalian expression of ATF **1–4** and Aart$_6$.

	Amino Acid Sequence
ATF **1**	MRSSKNVIKEFMRFKVRMEGTVNGHEFEIEGEGEGRPYEGHNTVKLKVTKGGPLPFAWDILS<br>PQFQYGSKVYVKHPADIPDYKKLSFPEGFKWERVMNFEDGGVVTVTQDSSLQDGCFIYKVKF<br>IGVNFPSDGPVMQKKTMGWEASTERLYPRDGVLKGEIHKALKLKDGGHYLVEFKSIYMAKK<br>PVQLPGYYYVDSKLDITSHNEDYTIVEQYERTEGRHHLFLGSGEGRGSLLTCGDVEENPGPLE<br>PGEKPYKCPECGKSFSTTGNLTVHQRTHTGEKPYKCPECGKSFSSRRTCRAHQRTHTGEKPYK<br>CPECGKSFSQKSSLIAHQRTHTGEKPYKCPECGKSFSQRANLRAHQRTHTGEKPYKCPECGKS<br>FSRSDELVRHQRTHTGEKPYKCPECGKSFSTTGALTEHQRTHTGKKTSPKKKRKVEASGSGR<br>ADALDDFDLDMLGSDALDDFDLDMLGSDALDDFDLDMLGSDALDDFDLDMLYPYDVPDYA
ATF **2**	MRSSKNVIKEFMRFKVRMEGTVNGHEFEIEGEGEGRPYEGHNTVKLKVTKGGPLPFAWDILS<br>PQFQYGSKVYVKHPADIPDYKKLSFPEGFKWERVMNFEDGGVVTVTQDSSLQDGCFIYKVKF<br>IGVNFPSDGPVMQKKTMGWEASTERLYPRDGVLKGEIHKALKLKDGGHYLVEFKSIYMAKK<br>PVQLPGYYYVDSKLDITSHNEDYTIVEQYERTEGRHHLFLGSGEGRGSLLTCGDVEENPGPLE<br>PGEKPYKCPECGKSFSSPADLTRHQRTHTGEKPYKCPECGKSFSSPADLTRHQRTHTGEKPYK<br>CPECGKSFSTHLDLIRHQRTHTGEKPYKCPECGKSFSQNSTLTEHQRTHTGEKPYKCPECGKSF<br>STHLDLIRHQRTHTGEKPYKCPECGKSFSQSSNLVRHQRTHTGKKTSPKKKRKVEASGSGRAD<br>ALDDFDLDMLGSDALDDFDLDMLGSDALDDFDLDMLGSDALDDFDLDMLYPYDVPDYA
ATF **3**	MRSSKNVIKEFMRFKVRMEGTVNGHEFEIEGEGEGRPYEGHNTVKLKVTKGGPLPFAWDILS<br>PQFQYGSKVYVKHPADIPDYKKLSFPEGFKWERVMNFEDGGVVTVTQDSSLQDGCFIYKVKF<br>IGVNFPSDGPVMQKKTMGWEASTERLYPRDGVLKGEIHKALKLKDGGHYLVEFKSIYMAKK<br>PVQLPGYYYVDSKLDITSHNEDYTIVEQYERTEGRHHLFLGSGEGRGSLLTCGDVEENPGPLE<br>PGEKPYKCPECGKSFSDPGHLVRHQRTHTGEKPYKCPECGKSFSRSDELVRHQRTHTGEKPYK<br>CPECGKSFSRADNLTEHQRTHTGEKPYKCPECGKSFSQSGDLRRHQRTHTGEKPYKCPECGKS<br>FSRRDELNVHQRTHTGEKPYKCPECGKSFSQLAHLRAHQRTHTGKKTSPKKKRKVEASGSGR<br>ADALDDFDLDMLGSDALDDFDLDMLGSDALDDFDLDMLGSDALDDFDLDMLYPYDVPDYA
ATF **4**	MRSSKNVIKEFMRFKVRMEGTVNGHEFEIEGEGEGRPYEGHNTVKLKVTKGGPLPFAWDILS<br>PQFQYGSKVYVKHPADIPDYKKLSFPEGFKWERVMNFEDGGVVTVTQDSSLQDGCFIYKVKF<br>IGVNFPSDGPVMQKKTMGWEASTERLYPRDGVLKGEIHKALKLKDGGHYLVEFKSIYMAKK<br>PVQLPGYYYVDSKLDITSHNEDYTIVEQYERTEGRHHLFLGSGEGRGSLLTCGDVEENPGPLE<br>PGEKPYKCPECGKSFSDKKDLTRHQRTHTGEKPYKCPECGKSFSQSSSLVRHQRTHTGEKPYK<br>CPECGKSFSQRAHLERHQRTHTGEKPYKCPECGKSFSRKDNLKNHQRTHTGEKPYKCPECGK<br>SFSQRANLRAHQRTHTGEKPYKCPECGKSFSQKSSLIAHQRTHTGKKTSPKKKRKVEASGSGR<br>ADALDDFDLDMLGSDALDDFDLDMLGSDALDDFDLDMLGSDALDDFDLDMLYPYDVPDYA
Aart$_6$	MASSEDVIKEFMRFKVRMEGSVNGHEFEIEGEGEGRPYEGTQTAKLKVTKGGPLPFAWDILSP<br>QFQYGSKVYVKHPADIPDYKKLSFPEGFKWERVMNFEDGGVVTVTQDSSLQDGSFIYKVKFI<br>GVNFPSDGPVMQKKTMGWEASTERLYPRDGVLKGEIHKALKLKDGGHYLVEFKSIYMAKKP<br>VQLPGYYYVDSKLDITSHNEDYTIVEQYERAEGRHHLFLGSGEGRGSLLTCGDVEENPGPISEF<br>GSSSSVAQAALEPGEKPYACPECGKSFSRSDHLAEHQRTHTGEKPYKCPECGKSFSDKKDLTR<br>HQRTHTGEKPYKCPECGKSFSQRANLRAHQRTHTGEKPYACPECGKSFSQLAHLRAHQRTHT<br>GEKPYKCPECGKSFSREDLNHTHQRTHTGEKPYKCPECGKSFSRRDALNVHQRTHTGKKTSP<br>KKKKKVEASGSGRADALDDFDLDMLGSDALDDFDLDMLGSDALDDFDLDMLGSDALDDFD<br>LDMLYPYDVPDYA

Supplementary Table 5. Amino acid sequences for the subcomponents of ATF **1–4**.

Component	Amino Acid Sequence
DsRed	MRSSKNVIKEFMRFKVRMEGTVNGHEFEIEGEGEGRPYEGHNTVKLKVTKGGPLPFA<br>WDILSPQFQYGSKVYVKHPADIPDYKKLSFPEGFKWERVMNFEDGGVVTVTQDSSLQ<br>DGCFIYKVKFIGVNFPSDGPVMQKKTMGWEASTERLYPRDGVLKGEIHKALKLKDGG<br>HYLVEFKSIYMAKKPVQLPGYYYVDSKLDITSHNEDYTIVEQYERTEGRHHLFL
GSG linker	GSG
T2A	EGRGSLLTCGDVEENPGP
NLS	PKKKRKV
VP64	DALDDFDLDMLGSDALDDFDLDMLGSDALDDFDLDMLGSDALDDFDLDML
HA tag	YPYDVPDYA

Supplementary Table 6. Amino acid sequence for SUMO-ATF **3r**.

	Amino Acid Sequence[1]
SUMO-ATF **3r**	MGSSHHHHHHGSGLVPRGSASMSDSEVNQEAKPEVKPEVKPETHINLKVSDGSSEI<br>FFKIKKTTPLRRLMEAFAKRQGKEMDSLRFLYDGIRIQADQTPEDLDMEDNDIIEAH<br>REQIGGMLEPGEKPYKCPECGKSFSDPGHLVRHQRTHTGEKPYKCPECGKSFSRSDE<br>LVRHQRTHTGEKPYKCPECGKSFSRADNLTEHQRTHTGEKPYKCPECGKSFSQSGD<br>LRRHQRTHTGEKPYKCPECGKSFSRRDELNVHQRTHTGEKPYKCPECGKSFSQLAH<br>LRAHQRTHTGKKTSPKKKRKVEASGSGRADALDDFDLDMLGSDALDDFDLDMLG<br>SDALDDFDLDMLGSDALDDFDLDMLYPYDVPDYA

[1] Underlined sequence indicates the SUMO tag which is cleaved by Ulp-1.

Supplementary Table 7. Nucleotide sequences for of SUMO-ATF **3r**.

	Nucleotide Sequence[1]
SUMO-ATF 3**r**	AATAATTTTGTTTAACTTTAAGAAGGAGATATACATATGGGCAGCAGCCATCAT<br>CATCATCATCACGGCAGCGGCCTGGTGCCGCGCGGCAGCGCTAGCATGTCGGAC<br>TCAGAAGTCAATCAAGAAGCTAAGCCAGAGGTCAAGCCAGAAGTCAAGCCTGA<br>GACTCACATCAATTTAAAGGTGTCCGATGGATCTTCAGAGATCTTCTTCAAGAT<br>CAAAAAGACCACTCCTTTAAGAAGGCTGATGGAAGCGTTCGCTAAAAGACAGG<br>GTAAGGAAATGGACTCCTTAAGATTCTTGTACGACGGTATTAGAATTCAAGCTG<br>ATCAGACCCCTGAAGATTTGGACATGGAGGATAACGATATTATTGAGGCTCACA<br>GAGAACAGATTGGTGGTATGCTGGAACCAGGGGAAAAACCATACAAATGCCCA<br>GAGTGCGGAAAGTCATTTTCGGACCCCGGTCACCTGGTCCGCCACCAACGGACA<br>CATACGGGCGAAAAGCCCTATAAGTGCCCTGAATGTGGCAAAAGTTTCTCGAGA<br>TCCGATGAGCTTGTTCGGCACCAACGTACGCACACTGGAGAAAAACCTTACAAA<br>TGTCCTGAGTGCGGTAAATCATTCAGTCGGGCGGACAACCTGACAGAGCATCAG<br>AGAACTCACACTGGAGAGAAGCCATATAAATGCCCGGAATGTGGTAAGTCTTTT<br>TCTCAATCTGGTGATTTACGTCGTCATCAGCGCACGCATACGGGTGAGAAGCCT<br>TATAAGTGCCCCGAATGCGGCAAGAGCTTTTCCCGGCGGGACGAGTTGAACGTG<br>CACCAACGGACCCACACAGGAGAAAAACCGTATAAGTGCCCGGAGTGCGGTAA<br>GAGTTTTTCCCAACTTGCACATCTTCGCGCTCATCAGCGTACCCATACTGGTAAG<br>AAGACAAGCCCCAAGAAGAAGAGGAAGGTGGAGGCCAGCGGTTCCGGACGGG<br>CTGACGCATTGGACGATTTTGATCTGGATATGCTGGGAAGTGACGCCCTCGATG<br>ATTTTGACCTTGACATGCTTGGTTCGGATGCCCTTGATGACTTTGACCTCGACAT<br>GCTCGGCAGTGACGCCCTTGATGATTTCGACCTGGACATGCTGTACCCCTACGA<br>TGTACCGGATTACGCTTGATAAA

[1] Underlined sequence indicates the SUMO tag which is cleaved by Ulp-1.
